# War, mental health and the emergence of ‘geopsychiatry’

**DOI:** 10.1192/bji.2025.10044

**Published:** 2025-08

**Authors:** Hussien Elkholy

**Affiliations:** Clinical Lecturer in Psychiatry, Department of Clinical Neuroscience, Brighton and Sussex Medical School, Brighton, UK.

It is almost impossible to watch the news nowadays without hearing a mention of a devastating situation somewhere on the globe. Whether due to natural disasters or man-made, the focus has been expanding over the past few years beyond physical consequences and infrastructural damage, to include also the profound psychological impact on mental health. Forced displacement, conflict-related trauma and chronic uncertainty contribute to the mental health burden among affected populations and the services dealing with it.^1^

Recently, ‘geopsychiatry’ emerged as a new subdiscipline in psychiatry, focusing on geopolitical factors and their effect on the mental health of individuals and communities. This includes political, geographical, commercial, cultural, economic and other factors that are often ignored in the context of causation of psychiatric disorders and deserve much better recognition in research, healthcare provision and policy-making.^2,3^

In the current issue we have two articles that fall into the domain of geopsychiatry. Forcibly displaced people have often been exposed to or have witnessed significant violence and abuse and experienced homelessness, loss of belongings, separation from family and friends, social and economic hardships, poor nutrition and lack of healthcare in their countries of origin, during transit and on resettlement.^4^ The article by Lashwood et al explores the impact and the care needs of the increasing numbers of asylum seekers from the perspective of crisis mental health services, focusing on a crisis assessment and home treatment team in London, UK.^5^ The authors highlight factors that can affect asylum seekers’ access to services, including awareness, cultural attitudes to mental illness and treatments, and concerns about confidentiality, trust and impact on any asylum claim. They also consider service-based barriers, such as availability of culturally and linguistically informed information.

On the other hand, Farouki et al discuss a different approach, showcasing the experience of a tripartite international collaboration aimed at improving mental health services at the site of conflict.^6^ The article discusses the need for a collective and systemic approach that emphasises partnership, local ownership and sustainability in developing and implementing mental health strategies to address the long-standing difficulties faced by mental health services in Palestine and exacerbated by the political conflict.

These two articles, among others in the current issue, demonstrate efforts in different countries to improve mental health services. I hope you find this issue informative.
